# Perspectives on sleep of people living with dementia in nursing homes: a qualitative interview study

**DOI:** 10.1186/s12877-023-04052-4

**Published:** 2023-05-26

**Authors:** Jonas Dörner, Johann-Moritz Hüsken, Kathrin Schmüdderich, Claudia Dinand, Martin N. Dichter, Margareta Halek

**Affiliations:** 1 Deutsches Zentrum für Neurodegenerative Erkrankungen (DZNE), Stockumer st. 12, 58453 Witten, Germany; 2 School of Nursing Science, Faculty of Health, Witten/Herdecke University, Alfred- Herrhausen-st. 45, 58455 Witten, Germany; 3 German Institute of Applied Nursing Research, Hülchrather st. 15, 50670 Cologne, Germany; 4 Institute of Nursing Science, Medical Faculty and University Hospital of Cologne, University of Cologne, Gleueler st. 176-178, 50935 Cologne, Germany

**Keywords:** Dementia, Sleep, Sleep disturbances, Nursing home, Qualitative study, Thematic analysis

## Abstract

**Background:**

Disturbed sleep among people living with dementia in nursing homes is widespread and is associated with diseases and all-cause mortality. This study examined the sleep of people living with dementia from their perspectives in nursing homes and that of the nurses who care for people living with dementia.

**Methods:**

A qualitative cross-sectional study was conducted. A total of 15 people living with dementia and 15 nurses in 11 German nursing homes were enrolled in this study. Data was collected between February and August 2021 through semistructured interviews, which were audio recorded and transcribed. Thematic analyses were performed by three independent researchers. Thematic mind maps and controversial findings were discussed with the *Research Working Group of People with Dementia* of the German Alzheimer Association.

**Results:**

Thematic analysis identified five overarching themes from the nursing home participants regarding sleep patterns: (1) characteristics of good sleep, (2) characteristics of bad sleep, (3) personal influences of people living with dementia on sleep, (4) environmental factors on sleep, and (5) sleep strategies of people living with dementia. Analysis also identified five overarching themes from the nurses participants: (1) characteristics of good sleep, (2) characteristics of bad sleep, (3) personal influences on sleep, (4) environmental factors on sleep, and (5) interventions for sleep promotion.

**Conclusions:**

The thematic analyses demonstrated that the perspectives of people living with dementia and nurses indicate the need to give more consideration to psychosocial factors and individual aspects of sleep in clinical practice. The results could also be helpful for the development of targeted assessment instruments and complex non-pharmacological interventions to promote sleep.

**Supplementary Information:**

The online version contains supplementary material available at 10.1186/s12877-023-04052-4.

## Background

Dementia is characterized as a syndrome that leads to a decline in cognition and affects independent daily functioning [1]. The number of people living with dementia (PLWD) worldwide was 57 million in 2019 and is expected to increase approximately 40% to 157 million in 2050 [2]. A systematic review of residents in the nursing home setting indicated an average prevalence of dementia of 58% [3].

In general, sleep has a huge impact on health and quality of life [4]. Many PLWD suffer sleep disturbances that keep them from obtaining of sleep, with an occurrence of up to 38% in nursing home populations [5, 6]. Disturbed sleep in PLWD, is associated with different diseases, neuropsychiatric symptoms and risks, such as depression, disinhibition, aberrant motor behaviour [7] and all-cause mortality [8].

Moreover, sleep disturbances in PLWD are a challenge for nursing home staff. A recent cross-sectional study in Germany showed that sleep disturbances in PLWD are a common problem for nurses working nightshifts. Approximately four out of five nurses are regularly confronted with residents demonstrating disturbed sleep and indicating moderate to severe emotional distress [6]. Furthermore, the care dependency on day and night changes significantly over time [9].

Sleep medications are commonly used even if they do not have a clear effect on PLWD [10]. Therefore, National guidelines recommend the use of non-pharmacological interventions as the first choice [11] and discourage pharmacological treatment due to a lack of high-grade evidence [12]. Systematic reviews have demonstrated that there are several non-pharmacological interventions available and that, complex non-pharmacological interventions seem to have the highest potential to be effective [13, 14]. However, thus far, there is no clear evidence of an effect, and there are still many unanswered questions regarding the mechanism of action [14].

There has been no investigation of the important aspects of non-pharmacological interventions and mechanisms of action from the perspectives of PLWD as a target group and nurses as important stakeholders, although recommended as part of the development of complex interventions [15].

The aim of the present study was to examine the perspectives on sleep of PLWD in nursing homes and nurses working in nursing homes to understand how this knowledge could contribute to improving sleep. The research question as follows: How is sleep experienced by PLWD and nurses in nursing homes and which factors influence sleep from their perspectives?

## Methods

The qualitative research design and thematic analysis were chosen to investigate experiences, meanings and the reality of participants [16]. We designed a qualitative cross-sectional interview study, that was embedded within the study *“*Multimodal non-pharmacological intervention for sleep disturbances in nursing home residents with dementia: A cluster-randomized controlled exploratory trial” (MoNoPol-Sleep) [17].

### Ethical considerations

All participants provided written informed consent, and ongoing consent was considered within interviews of PLWD [18]. No PLWD or nurses dropped out after signing informed consent. The ethical committee of the German Society of Nursing Science approved the study design (no. 20 − 016).

### Sample and recruitment

Two purposive samples [19, 20] of PLWD and nurses were selected that were most likely to contain the needed information to answer the research question. PLWD (in nursing homes, with a dementia diagnosis or possible dementia and disturbed sleep) and nurses (age, professional education, sex and work experience) were selected from 11 German nursing homes. Recruitment of study participants was carried out with the support of the nursing home manager or nurses from the nursing homes participating in the MoNoPol-Sleep trial [17]. Each nursing home was asked for a maximum of three participants (PLWD and nurses) to achieve heterogeneous samples related to nursing homes. Furthermore, four nurses were recruited from three additional nursing homes where the authors had conducted successful research projects in the past. The reason for nonparticipation was the nursing home manager’s lack of time to introduce the study to nurses or PLWD, or the nurses’ lack of time or the lack of ability of PLWD to participate in an interview (according to the nursing home manager’s judgement). None of the participants were reimbursed for study participation.

PLWD were eligible to participate in the study with diagnosed dementia or a Dementia Screening Scale (DSS) [21] score of ≥ 3. The DSS is a proxy assessment instrument that contains seven items. The total score varies between 0 and 14 (higher scores indicate higher cognitive impairment). The presence of at least two sleep problems according to the Sleep Disorders Inventory (SDI) [22] was a further inclusion criterion. The SDI is a proxy assessment instrument that contains seven items. The total score varies between 0 and 12 (higher scores indicate stronger sleep disturbances). Moreover, PLWD were required to have lived at least two weeks in the nursing home. PLWD were excluded if they had a diagnosis of sleep apnoea or rapid eye movement (REM) sleep disorder or if they received respite care in the nursing home. Furthermore, nursing assistants were excluded.

The, criteria for nurses was as follows: at least three years of professional education- which is the standard in Germany for registered nurses, three or more nightshifts within the last three months and an employment contract of ≥ 50% of the regular weekly working hours.

### Data collection

For each target group (PLWD and nurses), a semistructured interview guide was developed (see additional file 2). The development process was guided by the research question, available literature [23] and several discussion rounds in the research group. Within the discussion rounds, MH and MND served as supervisors for the qualitative study, and CD served as an external expert for qualitative research on dementia. No relationships between the participants and the interviewer existed before the study started, and the pseudonymity for PLWD and nurses was randomly assigned through a research data manager who was responsible for the organization of the data and was not involved in the field study. Data collection took place in August 2021 with PLWD and from February to June 2021 with nurses. After the first interview, the order of questions was slightly adapted for the final version (PLWD and nurses) to ensure a better interview flow. Before the recording of each interview started, as a kind of warm-up phase, information about the study was given, and the written consent that was verified in preparation for data collection was checked orally again. Within the interviews, the questions were repeated using different words if PLWD or nurses had difficulty understanding the original questions. After each interview, audio recording was switched off, and a context form was completed with important information, e.g., description of the setting, disruptive factors or additional notes, followed by a digital questionnaire for collection of sociodemographic characteristics. Interviews with all participants were conducted once. All interviews with nurses were collected via telephone interviews or online video meetings. The data for PLWD were all collected in the participating nursing homes of the MoNoPol-Sleep trial as face-to-face interviews. Two nurses attended five interviews with PLWD as a form of social support. All other interviews (for both PLWD and nurses) were held solely with the interviewer (JD).

In the course of data collection, the interview content was repeated for both target groups, and no new information emerged during these interviews. Therefore, after reaching 30 interviews, the data collection was closed (see criterion of dependability under the subheading trustworthiness).

### Data analysis

We conducted a thematic analysis according to Braun and Clarke [16] to achieve subjective perspectives on sleep. It contains six phases: (1) becoming familiar with the data, (2) conception of initial codes, (3) examining themes, (4) verifying themes, (5) defining and naming themes, and (6) generating the report. Additionally, thematic mind maps and controversial results were discussed in an online meeting with four PLWD (2 female, 2 male) and one employee of the *Research Working Group of People with Dementia* of the German Alzheimer Association.

For the analysis all data were audio recorded and later transcribed verbatim by a student assistant (PLWD interviews) and a transcription office (interviews with nurses). The first author verified all transcripts. None of the transcripts or findings were returned to participants for correction or comments. For both samples, two independent researchers open coded and analysed the data (PLWD: JD and KS, nurses: JD and JMH) with MAXQDA 2022 software. After all interviews were completely analysed, two additional steps were conducted: (1) discussing the two data sets separately within the assigned researchers for the qualitative analysis and (2) discussing the two data sets together within the research team. If disagreement occurred, CD was included as an external supervisor. The final versions of the thematic analysis were finally discussed with MH and MND.

Trustworthiness

To ensure trustworthiness, the criteria of Lincoln and Guba [24] were applied. The whole research process is transparent, as demonstrated within the article and with the use of an elaborated reporting guideline [25] (criterion of credibility). Moreover, when the investigation is repeated, we expect nearly the same themes (criterion of dependability). Because of the analysis by three independent researchers (see data analysis and authors’ information), congruence is given (criterion of confirmability). The identified themes are transferable for PLWD in German nursing homes (criterion of transferability). However, there could be some differences at the subtheme level depending on, e.g., the characteristics of the target group, health care settings and cultures.

## Results

### Participants

A total of 15 PLWD out of six nursing homes and 11 nurses out of 8 nursing homes were successfully recruited (Table 1). The mean duration of the interviews was 7 min (4–13 min) for PLWD and 43 min (26–85 min) for nurses.

**Table 1:** Demographic characteristics of PLWD and nurses

People living with dementia	n = 15	Nurses	n = 15
Age, mean (range)	84 (73–96)	Age, mean (range)	42 (26–62)
Sex, |*x*|		Sex, |*x*|	
Female	14	Female	11
Male	1	Male	4
DSS, mean (range)	8 (3–13)	Employment contract, %, mean (range)	93 (50–100)
Diagnosed dementia, |*x*|	14	Working years as nurse, mean (range), |*x*|	15 (6–37)
Dementia specific ward, |*x*|	5	Mainly working at nightshift, |*x*|	12

DSS: Dementia Screening Scale

### Themes emerged from the analysis

The thematic analysis of interviews for PLWD revealed five overarching themes: (1) characteristics of good sleep, (2) characteristics of bad sleep, (3) personal influences of PLWD on sleep, (4) environmental factors on sleep, and (5) sleep strategies of PLWD. Five themes were identified for nurses: (1) characteristics of good sleep, (2) characteristics of bad sleep, (3) personal influences on sleep, (4) environmental factors on sleep, and (5) interventions for sleep promotion. For both thematic mind maps with all subthemes, see additional file 1. All chosen statements of PLWD and nurses that were used to explain the themes are direct quotes (not paraphrased). The first author performed the translation into English, and in a second step, all quotes were critically reviewed and approved by a native English speaker with excellent German language skills who is experienced in scientific translations and cultural adaptation.

### Themes that emerged from the perspectives of people living with dementia

#### Characteristics of good sleep

PLWD characterized a good night of sleep as not waking up during the night or having been able to sleep through the night and feel emotionally well the next morning.

*When I’ve slept well (…) how I can put it? (…) When I’ve slept well and am rested, then I feel good. (7C0B1DFB)*

#### Well (after sleep), that I am in a good mood. (0F9E0C3B)

Some PLWD mentioned the possibility to do a lot the next day and to feel like the person who they were in the past as indicators for good sleep.

#### Than I am able to do a lot. (876AD9C)

### Characteristics of bad sleep

The most commonly mentioned characteristics of bad sleep were restlessness and pondering about the past or the future. As a result, PLWD could not fall asleep and stay awake for a long time. Nightmares and the need to use the toilet often were also mentioned as disturbing barriers to sleep at night.

*Sometimes I lie awake for a long time, I don’t fall asleep because of pondering when I am in bed. (605D9716)*

In addition to aspects at night, poor wellbeing and reduced cognitive and physical performance during the next day were also frequently mentioned as characteristics of bad sleep. Furthermore, PLWD described getting up earlier than usual or, in contrast, difficulties getting out of bed, and being less responsive.

#### There it’s like your head is drunk then you stagger around somehow. (98FE91AB)

### Personal influences of PLWD on sleep

PLWD often struggled with the nursing home as a place to feel at home, which they mentioned in cases that would have had a negative influence on their sleep.

*Where? Preferably at home and home is no longer there. House is sold (…). Here I sleep very bad. (2C0C3534)*

Age and complaints such as pain or oedema had a negative impact on sleep. Some mentioned health in general as a factor for good sleep.

*(…) Then I wake up and when I feel pain (…) when nothing hurts I turn over and carry on sleeping. (C68A2B77)*

The personal attitude towards sleep and individual happenings over the day and classification of those happenings could have a positive influence on sleep.

#### It doesn’t matter. But I can definitely sleep well, no matter what’s going on. (A6C2659C)

*That depends how the day is going. If you feel well or if you don’t feel well. (211158CF)*

### Environmental factors on sleep

PLWD described the importance of having the opportunity to individualize the atmosphere of their room to better enable sleep. Important related factors included lighting, climatic conditions (temperature or weather), fresh air, comfort, an individual order, room furnishings and privacy. Nurses’ activities at night, such as incontinence care and standardized nursing home processes, such as being woken up at fixed times in the morning for breakfast, also influenced sleep.

*(…) It always has to be tidy, I am very precise. I could not think “Well, let’s tidy up the room tomorrow”. (DFC5CC56)*

Often, the influence of noise inside and outside of the nursing homes was mentioned. Other residents who were singing or shouting at night and noises from cars or dogs were disturbing. However, silence in the environment was indicated as a sign of good sleep.

*Because, yes how can I describe that to you? I am old now. Old, and then you hear such a lot. Most of the times just stupid noises. (B5CDD561)*

When asked what was needed for good sleep in the social environment, PLWD stated that family and friends were important factors. Knowing that the family is fine and being visited regularly at the same time was especially important for PLWD. Nurses were important for conveying the feeling of security at night.

*Yes, my family somehow, my sons. I have two sons or do I have three? No, two sons. (98FE91AB)*

### Sleep strategies of PLWD

Individual routines, such as the right time for dinner, the chosen time to go to bed, or presleep activities, e.g., taking a walk before sleep to calm down and avoid being upset, were mentioned as strategies that had a positive influence on sleep.

*Don’t go to bed too late (…). Yes, that is the most important thing. And don’t have dinner too late. (B0AF7FC5)*

When PLWD wake up during the night, they different options, such as getting up or using the toilet, and the knowledge that a nurse will respond to their call bell to meet their needs (e.g., food, drink, crying) was beneficial for sleep promotion.

*And then I go for a little walk so that I calm down again and everything is fine again. (A6C22659C)*

*When I had anything wrong, I just needed to ring the bell and a nurse would come to me, so there are no hindrances. (DF5CC56)*

### Themes that emerged from the perspectives of nurses

#### Characteristics of good sleep

Sleep patterns are viewed as not being disturbed when PLWD slept until the next morning without long waking phases, could return to sleep quickly and were able to wake or rise at their usual time. To assess good sleep during the night for PLWD, nurses used indicators such as a comfortable sleep position, relaxed facial expression and physiological parameters, such as breathing.

*I think that regular breathing is a clear sign that sleep is sound and that deep sleep takes place. (ECC4F580)*

In particular, good mood and calmness of PLWD after sleeping were viewed as signs of good sleep. Furthermore, nurses noticed that, after a good night of sleep, PLWD were in balance and boisterous during the day and were happy to see the staff. The ability to hold conversations was better when compared to nights with less sleep, and needs such as hunger and thirst were more easily expressed.

*Yes, from their mood after sleeping. When during the day or rather after sleeping they are in a good mood and friendly, you can actually assume that they have slept well. (S331876C0)*

#### Characteristics of bad sleep

Disturbed sleep was accompanied mainly by psychological difficulties, such as aggression, restlessness or fear. In contrast to indicators of good sleep, impulsive for activities such as a compulsiveness to clean, clear out the closet or move furniture were also expressed as signs of bad sleep. The running tendency for PLWD at night was prominently portrayed as a very stressful condition for nurses that is accompanied by shouting, knocking on doors or ringing of the alarm bell if PLWD leave the nursing home. The interviewed nurses reported that the next day can be difficult because the PLWD does not get out of bed in the morning or because increased daytime sleep occurs. The interviewees also observed that PLWD were retreating and/or rejecting food, care or social activities, and their cognitive receptivity was reduced compared to nights with sufficient sleep.

*And yes, she tells me that she can’t sleep or hasn’t slept. And you recognize it because she is eating less. So, the longer she has not slept for days, then she is simply eating less. She gets very unfriendly while being tended to. Maybe, she tend to be a little bit aggressive as well because she is so tetchy. (95150BCF)*

### Personal influences on sleep

Most often, the interviewees mentioned that sleep was individual and was linked to habits and rituals. Their background regarding their past professional life or experienced traumata and complaints were influencing factors of bad sleep.

*Yes, that is (laughs) such a super-individual thing. Some want to have the television switched on, (…). For some, the tiniest noise is already totally disruptive and they wake up. That’s why we are trying to keep this very individual. (D6102F69)*

The interviewed nurses described sleep patterns that have an influence on sleep in general, which were mainly associated with the times that PLWD typically go to bed or wake up and start the day. A problem was identified regarding feeling at home within the nursing home. PLWD wanted to return to their former home, and that problem arose especially at night because of the missing diversions. Sleep disturbance occurred because of the loss of home.

*(…) he is very unsatisfied at the moment because he would rather be at home. (61495FFA)*

### Environmental factors on sleep

Most nurses mentioned that noises and care activities at night have a strong influence on sleep. Nursing home structured activities, such as dinner time or bringing residents to bed too early because of time pressure, were expressed as barriers to good night-time sleep. Another often mentioned factor in the environment involved conditions outside of the nursing home, such as parties in the neighbourhood or an airport nearby, that could not be influenced by the staff. A full moon was reported as a trigger for disturbed sleep. In the social environment, few nurses reported a negative impact on sleep when family and friends came to visit or pick up PLWD to take to activities outside the nursing homes, such as dinner, because afterwards PLWD were emotionally agitated.

*In the surroundings – well, l actually – I would say the working utensils. If someone goes with the care trolley along the corridor, if the trolley is quiet or the wheels are somehow wobbling back and forth, that’s when they make more noise; then, cupboard doors, room doors, that they are sufficiently isolated when opened or rather oiled, that they don’t make a noise when opening, don’t squeak or bang when you close them. (S331876C0)*

*(…) but what I have noticed is that, many don’t believe in it but I think and I know, if people, or anybody, whether dement [they have dementia] or not, they react on full moon. (…). I know that studies say that this is not true. But then they should come into a nursing home. It’s true. It’s true. (70998AF9).*

### Interventions for sleep promotion

In summary, the theme of interventions for sleep promotion contains a large number of possibilities. The one that was mentioned most often was to let PLWD do things when their sleep is disturbed and not try to convince or force them into bed.

Since we let her do it, she is actually more stable the whole time. (31A37001)

*Better let her go, I’d say, just watch that she does not enter the rooms of other residents but don’t bring her back to bed, because that has always made it a bit worse. (C0DCCB36)*

Emotional accompaniment, e.g., conversations and a sense of security, was also expressed often as a way to support sleep promotion for PLWD.

*And when there is someone who says, I am here, I’m watching over you, I’ll take care of everything, you can sleep now without any worries, then- then they sleep well. (6FF80A62)*

Person oriented offers in individual cases or testing assumptions regarding what could help to promote sleep in if nothing is known about preferences of PLWD was often reported.

*I have, for example, a resident suffering from itching. It is clarified that there is nothing wrong with the kidneys, which can often be the reason. Nevertheless, I think, if something is itching her, what would we do if we were in this situation? You scratch yourself. So I scratch her. (ECC4F580)*

## Discussion

This study identified five themes for PLWD: (1) characteristics of good sleep, (2) characteristics of bad sleep, (3) personal influences of PLWD on sleep, (4) environmental factors on sleep, and (5) sleep strategies of PLWD and five themes for nurses: (1) characteristics of good sleep, (2) characteristics of bad sleep, (3) personal influences on sleep, (4) environmental factors on sleep, and (5) interventions for sleep promotion.

### Characteristics of good sleep

From the perspective of PLWD, good sleep is mostly linked to sleeping through the night, being in a good mood upon waking and being able to recall their physical abilities. Likewise, nurses rated sleeping through the night, emotional status the next morning and bodily abilities as important indicators of good sleep. Additionally, it is possible for nurses to obtain an impression of sleep quality for PLWD when they make a clinical check of the patients (e.g., body signs such as calm breathing). Hermann and Flick (2011) investigated resources for good sleep and found that important prerequisites for good sleep are calmness and daily activities, among others. Another aspect in the context of physical activities was investigated with respect to the reduction in daytime sleep [26]. In intervention studies, psychosocial actions by staff to promote calmness [27] and physical activities [28, 29] are essential components. Based on these results, it seems that calmness and physical activity are key drivers of having a good night of sleep and that PLWD experiencing calmness and engaging in physical activities offered by the nursing home during the day are also consequences of a good night of sleep, since both affect each other.

### Characteristics of bad sleep

Poor sleep was mainly characterized by PLWD as pondering and restlessness, similar to the description of the interviewed nurses, who observed aggression and fear as main characteristics in addition to restlessness. Interviewees (both PLWD and nurses) reported that the days after a bad night of sleep had negative consequences for the PLWD, such as a decline in the physical and cognitive abilities of the PLWD and not getting out of bed in the morning. The subthemes “not getting out of bed” and “getting up earlier” after bad sleep are controversial findings in the thematic analysis of PLWD. In the discussion with the *Research Working Group of People with Dementia* of the Germany Alzheimer Society, participants stated that handling bad sleep the next day can differ greatly between people. To be disciplined and get up, no matter how the night was and rest later in the day or to stay longer in bed and have the chance for a good day afterwards, were explanations for both subthemes. The rejection of food, care and daytime activities was observed by the nurses. Interestingly, both the PLWD and nurses primarily described the consequences of bad sleep and not the properties of bad sleep per se. However, similar characteristics were found in a study that focused on the effect of sleep disturbances rated by care home staff [23]. Aggression and agitation after bad sleep were indicated as problems, but there was also an increased affinity for physical aggression and retraction of personal care. Daytime sleepiness and retreat of PLWD as indicated by wanting to go back to bed and not wanting to participate in activities were also mentioned. Cognition is also impaired after poor sleep, e.g., the ability of PLWD to communicate in their typical manner [23]. It might be helpful to combine these active and, above all, passive symptoms during sleep screening in nursing homes to improve the detection of sleep disturbances.

### Personal influences on sleep

Regarding personal influences, which are important for good sleep, the major subtheme for PLWD in our findings was to feel at home within the nursing home. This underpins the need for nurses to support PLWD in coping with their different life situations in the nursing home compared to their previous lives. Only one study about sleep in nursing home care presented similar results [30]; therefore, further research should investigate how to ensure that PLWD can feel at home as an intervention. The personal attitude regarding sleep in general and happenings over the day have a strong influence on how PLWD were able to sleep or rather how they started the night. Furthermore, it was mentioned by PLWD that physiological complaints such as pain or oedema have an impact on sleep as well. Nurses reported similar complaints and underlying conditions, such as being hungry or experiencing pain. A literature review [31] identified factors that contribute to sleep disturbances in nursing home residents, such as age-related changes. Furthermore, as reported in our study by PLWD, underlying conditions such as dementia, depression or primary sleep disorders and other comorbidities were identified as sleep disruptive factors [31].

Concerning personal influences on nurses, the individuality of sleep is strongly connected to the habits, rituals and background of PLWD. The importance of knowing each PLWD and using this knowledge to develop interpersonal connections is a key requirement for providing good quality night-time care. Simultaneously, Nunez et al. stated that limited interactions and knowledge of PLWD among the night staff due to the diversity and cultural differences of the team has an impact on sufficiently knowing the PLWD and being able to help provide good quality night-time care [32]. From this, it can be concluded that it is important to implement strategies to make individual preferences visible for all professionals who are responsible for the care of PLWD and find a way to address these needs in the same way through all care providers.

### Environmental factors on sleep

The theme environment also has an important impact on sleep. Noises and light at night were brought up frequently in both interview groups as a barrier to undisturbed sleep and were also found in other studies [33]. Moreover, a lack of sufficient bright light during the day influences the circadian rhythm and leads to sleep disturbances [31]. Therefore, reflection on noises that are produced by care staff in resident rooms when care activities take place at night, and noises produced in common areas and hallways in relation to nightshift could be a helpful and easy intervention to improve the sleep of PLWD. Furthermore, artificial light should be reduced on floors and, if possible, completely switched off in resident rooms. In addition, natural sunlight during the day is a key element in the preparation of good sleep and should be received by PLWD as much as possible. This includes multiple periods outside of the nursing home. The full moon was mentioned by a few nurses in our interviews as an impact factor for sleep disturbances. One nurse discussed within her interview that no scientific evidence [34] exists about the Moon as an influencing factor on sleep, yet she still suggested that it is a factor. When asked about the potential negative effect of a full moon on sleep, the members of the *Research Working Group of People with Dementia* had differing opinions. Two PLWD reported that their sleep is disturbed a few days before the full moon, independent of the brightness of the moonlight. Two PLWD reported that they do not notice any differences compared to other phases of the moon. However, for our interviewed PLWD, it is important to personalize their private room, e.g., with room furnishings, and give them privacy. Previous research highlighted the need to make active decisions for oneself regarding privacy [35]. PLWD reported that contact with family and friends throughout the day is important for good sleep as well. In contrast, few nurses reported a negative impact on sleep when relatives visited PLWD or did something with them outside of the nursing home. This controversy was confirmed by the *Research Working Group of People with Dementia* of the German Alzheimer Society, and participants imagined that it can influence sleep in a positive or negative way. According to the interviewed nurses, nocturnal care activities and nursing home structures were mentioned as barriers to sleep promotion in the environment. Check-ups and night care within PLWD rooms were identified as problematic in other studies [33, 36]. One recommendation to promote sleep is to reduce these routine checks in resident rooms [30]. The findings of our qualitative study suggest that a reduction in routine work processes outside of residents’ rooms in general in nursing homes at night might influence sleep quality positively. Therefore, institutional and cultural changes are needed to improve sleep in nursing home residents [31].

### Sleep strategies and interventions to promote sleep

To avoid sleep complaints, PLWD in our study reported using individual routines before sleeping, such as having dinner earlier and going to bed early. Personal routines, such as counting objects in the room to promote sleep, have been described in another study [36]. In our study, PLWD indicated that if sleep complaints exist at night, then they have the option to calm down, take a walk, or eat and drink as potential preparations to return to sleep. A Health Technology Assessment recommends that feeding people at night could be counterproductive because PLWD learn to eat at atypical times [37], which is opposite to our findings (mentioned from both interviewed groups), suggesting that nocturnal food can help to promote a return to sleep at night. However, while incorporating emotional accompaniment, the nurses’ first priority is to support the PLWD and to let them do what helps them fall asleep. Developing a personal connection with PLWD in nursing homes [32] and the need for emotional support [36] were also reported as main subthemes for care staff elsewhere. Nevertheless, 51.5% of participants in a study that investigated the sleep of nursing home residents with a self-questionnaire mentioned not having any self-care strategies to manage sleep disturbances [38]. In these cases, clear advice by nurses can contribute to empowering PLWD regarding sleep promotion.

### Strengths and Limitations

First, a strength of this study is the inclusion of PLWD and nurses as the affected target group and the main professional acting occupational group in this setting to obtain comprehensive information about sleep in nursing homes for PLWD. Second, a strength is the purposive sampling strategy in multiple nursing homes to achieve a wide range of information related to the research question.

This research has some limitations. First, in Germany, the procedures to diagnose dementia are not applied for each nursing home resident [39]. We therefore decided to include PLWD by using a Dementia Screening Scale that indicates probable dementia. Second, it could have been helpful to integrate more perspectives of nurses who worked mainly on dayshifts in the nursing home when sleep and sleep promotion are understood as responsibilities of both night and day shifts. The third limitation is the procedure of the interviews with the nurses. Due to contact restrictions during the COVID-19 pandemic, only online or telephone interviews with the nurses were performed. This context could have had an impact on the interviews because nurses could have felt more distanced and uncomfortable than they would have if face-to-face interviews had been utilized. The topic of sleep and sleep disturbances in PLWD is sensitive, and studies have shown that nurses feel burdened by those concerns. However, before the interviews started, the interviewer introduced himself and informed the nurses about the MoNoPol-Sleep project and the reason for the qualitative interview study to try to achieve an open and relaxed atmosphere. The fourth limitation is that none of the transcripts or findings were returned to participants for correction or comments. However, we discussed the results in a meeting with the *Research Working Group of People with Dementia* of the German Alzheimer Association. They found the results in were understandable and agreed with the findings. Participants mentioned that not all subthemes are dementia or nursing home specific but more burdensome with dementia in the combination of getting older.

## Conclusion

The thematic analyses demonstrated five dimensions to better understand how sleep is experienced by PLWD and nurses in nursing homes. Sleep promotion should be understood as a comprehensive task that unites a natural scientific understanding of sleep mechanisms and the psychosocial perspective with a strong focus on individual preferences. In particular, feeling at home was mentioned as a core element in both interviewed groups and should be a major goal for care providers in sleep promotion. Furthermore, our results showed that it is important to make individual preferences visible. For this, it can be concluded that, a detailed sleep anamnesis in nursing records which take into account the perspective of PLWD and the assessment by nurses is an effective instrument for person-centered care planning. Furthermore, our results indicate, that it is important for nurses to consider that sleep promotion does not end when the night shift ends in the morning; rather, it needs to be understood as an ongoing task that takes place during the day and night.

Further research is needed to gain more knowledge about the interplay of sleep characteristics, environmental factors and sleep strategies/interventions as identified by PLWD and nurses. Suitable for our research, a recent a large study in the UK started to deepen the understanding of sleep disturbances in the primary care of PLWD and people with mild cognitive impairment [40]. As a subsequent step, the results of our study could be integrated into complex assessment development and non-pharmacological intervention research.

## Electronic supplementary material

Below is the link to the electronic supplementary material.

Supplementary Material 1

Supplementary Material 2

## Data Availability

The data generated and analysed during this study are not publicly available and not available from the corresponding author to protect participant confidentiality.

## Declarations

### Authors’ information

JD (RN, MScN) is a registered nurse who has several years of experience in geriatric care. He has a master’s degree in nursing science and has previously conducted dementia research. JMH (RN, MScN) and KS (RN, MScN) are nurses by training and have experience in geriatric care and dementia research (including analysis of qualitative data). All steps regarding preparation, conduct and analysis until finalization of both thematic analyses were in close consultation with CD (RN, MScN). She has experience in geriatric care and as a qualitative researcher, especially in dementia research, with a broad repertoire of methods. MH (PhD, RN) and MND (PhD, RN) served as supervisors and are also experienced in empirical dementia research.

## List of abbreviations

DSS: Dementia Screening Scale
MoNoPol-Sleep: Multimodal non-pharmacological intervention for sleep disturbances in nursing home residents with dementia:A cluster-randomized controlled exploratory trial
PLWD: people living with dementia
SDI: Sleep Disorders Inventory
