# Supplementary material for: Perspectives on sleep of people living with dementia in nursing homes: a qualitative interview study
Source: BMC Geriatr. 2023 May 26;23:331. doi: 10.1186/s12877-023-04052-4 (PMC10214326; doi:10.1186/s12877-023-04052-4)
Supplement: Supplementary file 1 — Supplementary Material 1 [file 12877_2023_4052_MOESM1_ESM.docx]

## **Additional file I**

File name: perspectives_on_sleep_Additional_file_1_BMC_Geriatrics

File format: Microsoft Word document (.docx)

Title of data: Thematic mind maps: Perspectives on sleep of people living with dementia in nursing homes: A qualitative interview study

Description of the data: The thematic mind maps of PLWD and nurses present the described analyses in the manuscript.

## **Thematic mindmaps: Perspectives on sleep of people living with dementia living in nursing homes: A qualitative interview study**


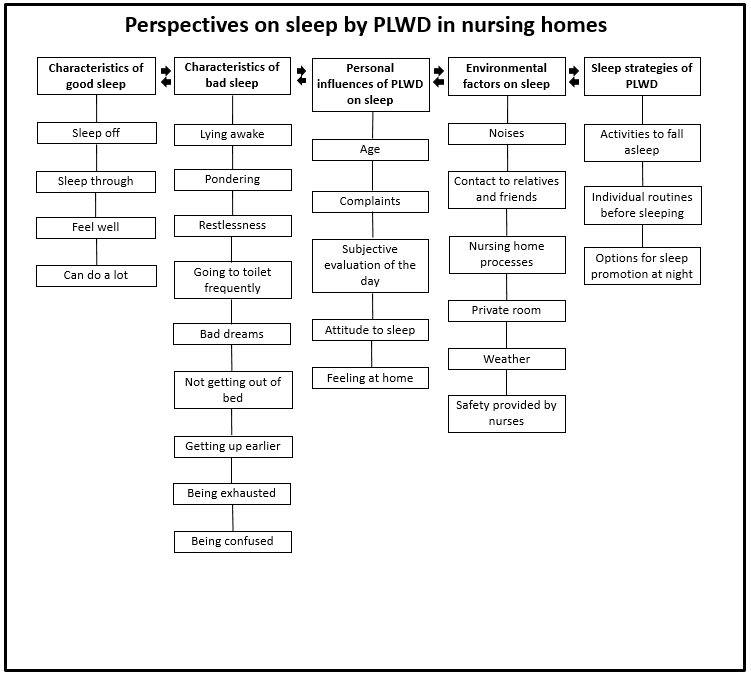
**Figure 1: Thematic mind map of PLWD**


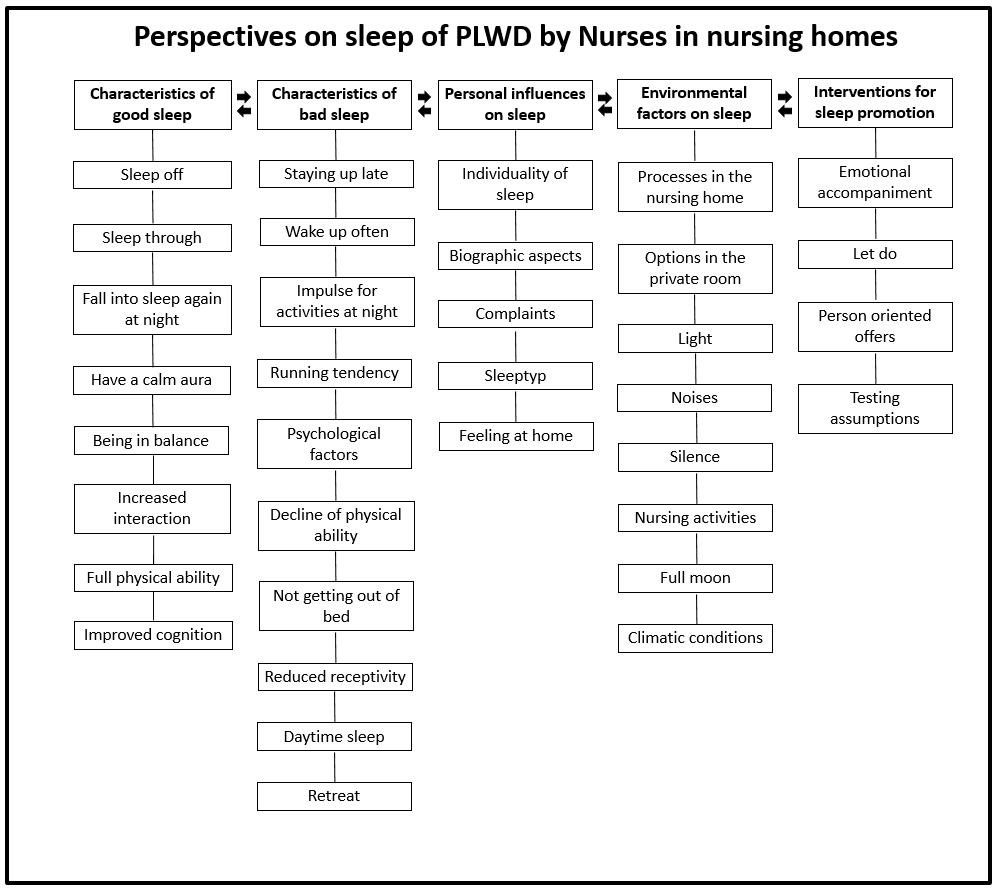


**Figure 2: Thematic mind map of nurses**
