# Supplementary material for: Perspectives on sleep of people living with dementia in nursing homes: a qualitative interview study
Source: BMC Geriatr. 2023 May 26;23:331. doi: 10.1186/s12877-023-04052-4 (PMC10214326; doi:10.1186/s12877-023-04052-4)
Supplement: Supplementary file 2 — Supplementary Material 2 [file 12877_2023_4052_MOESM2_ESM.docx]

## **Additional file 2:**

File name: perspectives_on_sleep_Additional_file_2_BMC_Geriatrics

File format: Microsoft Word document (.docx)

Title of data: Interview guides PLWD and Nurses: Perspectives on sleep of people living with dementia in nursing homes: A qualitative interview study

Description of the data: The interview guides for PLWD and nurses present the questions used in the interviews.

## **Interview guides PLWD and Nurses: Perspectives on sleep of people living with dementia in nursing homes: A qualitative interview study**

| **Interview guide people living with dementia** |
| --- |
| 1. **I would like to know, how do you sleep at the moment (perceive your sleep)?** 2. **Can you tell me, what does it mean for you to have had excellent sleep?** 3. **Can you tell me what is important for you to have excellent sleep?** 4. **Can you tell me, what does it mean for you to have slept very poorly?** 5. **Can you tell me the reasons why you slept very poorly?**   **Further (optional) questions**  **Is there something in your room that you need to sleep well?**  **Is there something that helps you to fall asleep easily?**  **Is there something that is important for you for a calm and comfortable night?**  **Is there something that disturbs you at night?**  **How many hours of sleep do you usually need to feel well rested?**  **Can you remember the last time you were not well rested? If yes, any reasons for that?**  **Final question: Is there anything else you would like to tell me that seems important regarding the topic of sleep?**  **Thank you very much for the interview.** |

| **Interview guide for nurses** |
| --- |
| **We are talking today about sleep disturbances in people living with dementia. Can you tell me, how do you perceive the sleep of people living with dementia in your nursing home?**  **Can you tell me, how do you recognize that a person with dementia had excellent sleep?**  **Can you tell me, how do you recognize that a person with dementia is sleeping very well?**  **Can you tell me, how do you recognize that a person with dementia slept very poorly?**  **Can you tell me, how do you recognize that a person with dementia is sleeping very poorly?**  **Sleep in detail (vignette study)**  **Now I would like to ask questions about two different residents with dementia who live in your nursing home. Imagine first a resident with sleep disturbances who suffered because of the sleep disturbances and second, a resident with sleep complaints who did not suffer.**  **Please tell me about the first special case or situation where a resident with dementia had sleep disturbances and suffered.**  **Can you describe the behaviour/symptoms?**  **Is there something that increases the behaviour/symptoms?**  **From the perspective of the resident: how do you think the resident has experienced the night? (Emotionally …)**  **What do you think was the cause of the sleep disturbances of the resident?**  **What have you done concretely during the night to solve the problem?**  **What else could you have done?**  **What must happen for the situation to be perceived as "better“?**  **What must happen for the situation to be perceived as "worse“?**  **What are the experienced consequences of the sleep disturbances for you and your work at night?**  **What are the experienced consequences for your colleagues?**  **What are the experienced consequences for other residents?**  **How long have you known the resident?**  **Has the sleep of the resident changed since you have known him or her?**  **If yes, how did the sleep of this resident change?**  **Is there something that could be responsible for these changes?**  ______  **Now, please tell me about the second case: a situation where a resident with dementia had sleep disturbances and, in your opinion, did not suffer.**  **Can you describe the behaviour/symptoms?**  **Is there something that enhanced the behaviour/symptoms?**  **From the perspective of the resident: how do you think the person has experienced the night? (Emotionally…)**  **What do you think was the cause of the sleep disturbances of the resident?**  **What have you done concretely this night to solve the problem?**  **What else could you have done?**  **What must happen for the situation to be perceived as "better“?**  **What must happen for the situation to be perceived as "worse“?**  **What are the consequences of the sleep disturbances for you and your work at night?**  **What are the consequences for your colleagues?**  **What are the consequences for other residents?**  **How long have you known the resident?**  **Did the sleep of the resident change since you have known him or her?**  **If yes, how have the sleep disturbances changed?**  **Is there something that could be responsible for the changes?**  ______  **What seems to be the difference between the first and the second case?**  **Further (optional) questions**  **How often during a night do you check if a resident is sleeping?**  **Do you know if this disturbs their sleep?**  **Please tell me, what components of the environment of a nursing home are important for a good night of sleep?**  **Can you describe what support you have at work when you help people living with dementia to go to bed in the evening?**  **Can you describe what might disturb the sleep of a resident in your nursing home at night?**  **Which factors are likewise playing an important role when we are thinking about sleep?**  **How do you evaluate how residents with dementia slept in the week before you start your night shift?**  **How do you evaluate the effect of sleep medication in general?**  **Final question: Is there anything else you would like to tell me that is important regarding the topic of sleep, and is there something that we have not talked about yet?**  **Thank you very much for the interview.** |
